# Sol Gel-Derived SBA-16 Mesoporous Material

**DOI:** 10.3390/ijms11093069

**Published:** 2010-08-31

**Authors:** Eric M. Rivera-Muñoz, Rafael Huirache-Acuña

**Affiliations:** 1 Centro de Física Aplicada y Tecnología Avanzada, Universidad Nacional Autónoma de México, A.P. 1-1010 Querétaro, Qro., Mexico, C.P. 76000, México; 2 Facultad de Ingeniería Química, Universidad Michoacana de San Nicolás de Hidalgo, Ciudad Universitaria, Morelia, 58060, México; E-Mail: rafael_huirache@yahoo.it

**Keywords:** SBA-16, mesoporous, sol gel derived, silica, template

## Abstract

The aim of this article is to review current knowledge related to the synthesis and characterization of sol gel-derived SBA-16 mesoporous silicas, as well as a review of the state of the art in this issue, to take stock of knowledge about current and future applications. The ease of the method of preparation, the orderly structure, size and shape of their pores and control, all these achievable through simple changes in the method of synthesis, makes SBA-16 a very versatile material, potentially applicable in many areas of science and molecular engineering of materials.

## 1. Introduction

Porous materials have attracted the interest of scientists and industry due to their potential applications, for instance in adsorption technology, molecular separation, catalysis, electronics, gas sensors, etc. as well as to the new challenges in the materials research field. Mesoporous materials were developed decades ago. Pillared clays, which possess mesopore sizes, have been extensively investigated since the 1980s. However, their rectangular pores could not be fully opened, had a wide distribution of pore size and their arrangement was disordered [1].

Studies on ordered mesoporous materials have attracted extensive attention since the discovery of the M41S family of mesoporous silicas in 1992 [2,3]. These M41S materials, exhibiting high surface area and volume, were formed by following the sol-gel method with an ionic surfactant (cetyltrimethylammonium bromide) as template. In this kind of materials the average pore size extends over the micro-to mesopore range. In addition, the concept of “template” was first postulated in the synthesis of mesoporous silicate materials [1].

More recently, Zhao *et al*. reported the synthesis of a variety of mesoporous SBA-type silica materials (SBA = Santa Barbara), using non-ionic triblock copolymers as template [4,5]. This type of surfactant is very interesting, because it is easily separated, is nontoxic, biodegradable, and inexpensive [5]. The synthesis conducted with these surfactants usually occurs in low-pH solutions (pH ≈ 2) where the interaction occurs through an S^0^H^+^X^−^I^+^ mechanism (S^0^H^+^ being the surfactant hydrogen-bonded to a hydronium ion, X^−^ the chloride ion, and I^+^ the protonated silica) [6]. SBA-type mesoporous silicas exhibit large pore sizes (20–300 Å), thick pore walls, and high stability [7]. Among these SBA-type silica materials, SBA-16 is considered a very interesting mesostructure due to the fact it has a 3D cubic arrangement of mesopores corresponding to the *Im3m* space group. Triblock copolymers with relatively large poly(ethylene oxide) (PEO) chains, such as Pluronic F127, F108, and F98, can serve as templates, but templates with very long PEO chains, such as F88 (EO_104_PO_39_EO_104_) and F68 (EO_76_PO_29_EO_76_), are seldom used due to the strict conditions necessary to obtain high-quality SBA-16 [1]. In addition, the mesophase can be created using mixtures of Pluronic P123 and Pluronic F127 templates or in a ternary water, butanol and Pluronic F127 system.

The structure of SBA-16 can be described by a triply periodic minimal surface of I-WP (body centered, wrapped package) [8]. The mesophase might also be a triply periodic minimal surface. As suggested by electron crystallography studies [8], each mesopore is connected to eight neighboring mesopores. The size of the entrance from one mesopore to another is usually significantly smaller than the primary mesopore; and the desorption out of this kind of structure is dominated by the so-called pore blocking and by networking effects [9,10]. The open frameworks and tunable porosities endow SBA-16 mesoporous material with accessibility to metal ions and reagents. These characteristics are extremely important in the fields of catalysis, separation membranes, sensors, electronic devices, biology and nanotechnology.

## 2. Synthesis Mechanism

### 2.1. The Sol-Gel Process

The sol–gel method constitutes nowadays an important route to synthesize porous silica without involving the deployment of excessively high temperatures [11]; additionally, this experimental technique allows one to control, to a certain extent, the sizes of the solid particles constituting the silica aerogel or xerogel.

The pathways leading to porous ordered materials are very similar to the sol-gel process where the polymerization is carried out in an aqueous solution by adding a catalyst and a source of silica. The silica source is an important factor for the reaction conditions. Common molecular sources are alkoxysilanes like tetramethyl- and tetraethylorthosilicate or sodium silicate. Non-molecular silica source are, for example, already polymerized sol-gel materials which lead to non-homogeneous solutions [12]. The first step of polymerization is the formation of silanol groups by hydrolysis of the alkoxide precursors, the gel, in aqueous solution:

$$\equiv \text{Si}-\text{OR}+{\text{H}}_{2}\text{O}↔\text{Si}-\text{OH}+\text{ROH}$$

The polymerization occurs through water (oxolations) or alcohol (alcoxolations)-producing condensations:

$$\begin{array}{l} \equiv \text{Si}-\text{OH}+\text{HO Si}\equiv ↔\equiv \text{Si}-\text{O}-\text{Si}\equiv +{\text{H}}_{2}\text{O}\\ \equiv \text{Si}-\text{OH}+\text{RO}-\;\text{Si}\equiv ↔\equiv \text{Si}-\text{O}-\text{Si}+\text{ROH} \end{array}$$

### 2.2. The Template Pathway

**Figure 1:**
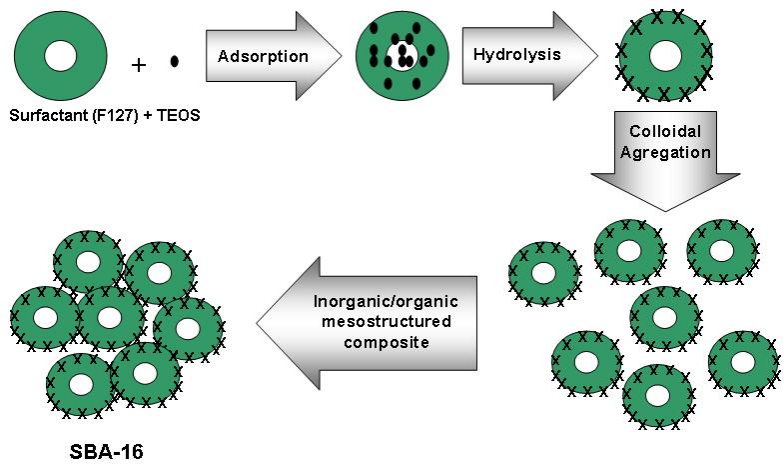
Schematic representation of the synthesis method of SBA-16.

The main wayto obtain a well defined and structured SBA-16 material is to use a surfactant templated polymerization instead of an uncontrolled reaction (Figure 1). The organic- inorganic self-assembly is driven by noncovalent weak bonds such as hydrogen bonds, van der Waals forces, and electrovalent bonds between the triblock copolymer surfactant and inorganic species. Instead of a simple superposition of the weak interaction, an integrated and complex synergistic reaction facilitates the process. Cooperative assembly between poly(ethylene oxide)-poly(propylene oxide)-poly(ethylene oxide), (EO_106_PO_70_EO_106_, Pluronic F127), organic surfactant and inorganic precursors is generally involved, forming inorganic/organic mesostructured composites. In general, the amphiphilic surfactant molecules form a liquid crystal by aggregation in aqueous solution [2,3]. The formation of the liquid crystal matrix strongly depends on the conditions in the solution. The structure of the liquid crystal is the so called mesostructure. Important parameters for the mesophase formation are for instance the temperature, concentration or the pH-value of the solution.

In order to act as a structure directing agent, the mesophase has to interact in some way with the silica precursors. There have been many different attempts to develop pathways to influence the interactions between mesophase and polycondensation reaction of the silica source. Stucky and co-workers have proposed four general synthetic routes, which are S^+^I^−^, S^−^I^+^, S^+^X^−^I^+^ and S^−^X^+^I^−^, (S^+^) surfactant cations, (S^−^) surfactant anions, (I^+^) inorganic precursor cations, (I^−^) inorganic precursor anions, (X^+^) cationic counterions, and (X^−^) anionic counterions [13]. On the other hand, hydrogen-bonding interaction mechanisms, namely, S^0^I^0^ or N^0^I^0^, were proposed by Pinnavaia and co-workers for preparing mesoporous silicates under neutral conditions [14,15]. S^0^ are neutral amines, N^0^ are nonionic surfactants, and I^0^ are hydrated silicate oligomers from tetraethyl orthosilicate (TEOS). As mentioned before, the non-ionic routes templated with amphiphilic triblock copolymers are used to synthesize the SBA-16 material. These routes are relatively new and have shown a high flexibility in tailoring the synthesis conditions and the mesostructure of the liquid crystal template.

### 2.3. Removal of Template

The porosity can only be obtained after the removal of template from the as-synthesized inorganic-organic composite. Different removal methods certainly influence the characteristics of SBA-16 mesoporous materials. The most common method to remove the template is calcination due to the easy operation and complete elimination. Organic surfactants can be totally decomposed or oxidized under oxygen or air atmospheres [1]. The temperature programming rate should be low enough to prevent the structural collapse caused by local overheating. A two-step calcinations was adopted by Mobil scientists—the first 1 h under nitrogen to decompose surfactants and the following 5 h in air or oxygen to burn them out [3]. This complicated procedure was then simplified; the first calcination step under nitrogen can be substituted by low rate heating in air. Heating the as-synthesized SBA-16 material with a rate of 1–2 °C/min to 550 °C and keeping this temperature for 4–6 h can completely remove triblock copolymer templates. The calcination temperature should be lower than the stable temperature of the mesoporous materials and higher than 350 °C to totally remove PEO-PPO-PEO type surfactants. Higher calcination temperatures would lead to lower surface areas, pore volumes, surface hydroxyl groups and higher cross-linking degrees of mesoporous materials. The drawbacks of calcination are the non-recovery of surfactants and the sacrifice of surface hydroxyl groups.

**Figure 2:**
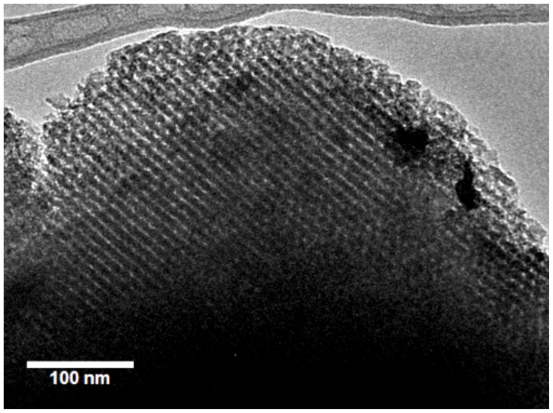
Typical HRTEM image of SBA-16 structure.

Extraction is a mild and efficient method to remove surfactants and to get porosities without distinct effects on frameworks [5,16]. Ethanol or THF can be used as an organic extracting agent. A small amount of hydrochloric acid is added in the extracting agent to improve the cross-linkage of frameworks and to minimize the effects on mesostructures [1]. With the aid of sulfuric acid, triblock copolymers in SBA-16 mesostructure can be removed [17–19], and tailored pore channels and structures can then be achieved. In addition, new procedures including microwave digestion [20,21] photo-calcination [22] as well as supercritical fluid extraction [23] were also applied and turned out to be beneficial for some ordered mesoporous materials. Figure 2 corresponds to a typical HRTEM image of SBA-16 in which both the structural order as well as the cubic symmetry of this material can be observed.

## 3. Synthesis Methods

SBA-16 can be synthesized under acidic conditions over a narrow range of dilute EO_106_PO_70_EO_106_ surfactant concentrations (3–5%) at room temperature (RT). After reacting at RT for 20 h, high-quality SBA-16 was produced by heating the solid precipitate in the mother solution at 80 °C for 2 days. Higher copolymer concentrations result in the formation of silica gel, while lower concentrations lead to the formation of amorphous silica [5]. The first report by Zhao *et al*. [5] is described as follows: in a typical preparation, 4.0 g of Pluronic F127 is dissolved in 30 g of water and 120 g of 2M HCl solution with stirring at RT. Then 8.50 g of tetraethyl orthosilicate (TEOS) is added into that solution with stirring for 20 h. The mixture is then aged at 80 °C overnight without stirring. The solid product is recovered, washed, and air-dried at RT. Yields are ~98% (based on silicon), which is comparable to the syntheses described above. Calcination is carried out by slowly increasing temperature from room temperature to 500 °C in 8 h and heating at 500 °C for 6 h [5].

To date the SBA-16 materials have been synthesized mostly by employing Pluronic F127 [4,5,24–30], however, there are a few reports on the use of copolymer blends [28] of P123 and F127 or even nonionic oligomeric surfactants [26] which led to small pore sizes. In addition, some interesting results were achieved when the synthesis was carried out by varying silica concentrations in the range of 0.75 to 1.2 in the final gel composition [25]. Furthermore, recent studies [27] have demonstrated that SBA-16 can be prepared even after one hour of stirring under highly acidic conditions. Recently, Li *et al*. [32] showed that the use of a mixed template consisting of Pluronic F127 copolymer and anionic surfactant sodium dodecyl sulfonate (SDS) afforded SBA-16 samples with smaller mesopores and enabled some possibility to control the microporosity in these materials by varying the SDS/F127 ratio. An analogous approach was also used by Mesa *et al*. [33] who instead of SDS employed cetyltrimethylammonium bromide (CTMABr) as co-surfactant. They proposed that the presence of cationic CTMABr can regulate the shape of micelles and their interaction with the silica precursors in the SBA-16 synthesis. In addition, CTABr was suggested to help control the morphology and regulate the shape of SBA-16 mesostructure [33–35]. However, Kleitz *et al*. [36] were able to obtain the SBA-16 mesostructure over a wide range of compositions of TEOS and Pluronic F127 at low acid concentration by using *n*-butanol as organic additive at low HCl concentration. However, limited SBA-16 synthesis methods have been reported because the cage-like SBA-16 mesostructured silica can only be produced in a narrow range of synthesis parameters [33–35,36,39]. SBA-16 single crystal (particle size ~ 1 ìm) was obtained under static condition by Yu *et al*. using block co-polymer F108 as template in presence of K_2_SO_4_ and HCl [37,38]. It was reported that the morphology and the particle size distribution are mainly determined by the regularity and the covering degree of the micelles by the silica species, by the poly-condensing degree (acidity and temperature), and by the Brownian movements (temperature) at the moment of the precipitation. The regularity of the structural arrangement and the porosity properties of the final mesoporous silica are likely also related to the same parameters [39].

## 4. Applications

### 4.1. Catalysts

The characteristics of SBA-16 material such as uniform pores and high surface area offer good opportunities in the catalysis field. High-quality single-walled carbon nanotubes (SWNTs) have been synthesized on SBA-16 type mesoporous silica thin film which provides an additional volumetric capacity for holding catalytic metals inside it [40–42]. In addition, multi-walled carbon nanotubes (MWNTs) have been prepared by catalytic chemical vapor deposition (CVD) method with Fe in SBA-16 as template [43]. On the other hand, SBA-16 and SBA-16-modified materials have been used as supports in order to obtain more active hydrodesulfurization (HDS) catalysts [44–47].

Chloroperoxidase (CPO) immobilization on functionalized SBA-16 mesoporous materials is an important application for this enzyme. In that work [48], Aburto *et al*. indicate that both the activity and the stability of the biocatalyst strongly depend on the immobilization method.

The encapsulation of homogeneous chiral catalysts, e.g., Co(Salen), [Ru(salen)(NO)] and Ru-TsDPEN, in the mesoporous cage of SBA-16 was demonstrated by different authors; the encapsulated catalysts showed performance as good as that of the homogeneous catalysts for the asymmetric transfer hydrogenation of different ketones and asymmetric ring opening of epoxides [49–53]. Yang, *et al*. [54] have prepared catalysts for enantioselective cyanosilylation of aldehydes by encapsulating a chiral vanadyl Salen complex [VO(Salen)] in the nanocage of SBA-16. In addition, SBA-16 thin films have been provided inside microreactors for use as a catalyst support and employed for a cyanosilylation reaction [55].

Monodispersed bimetallic Pt–Sn/SBA-16 catalysts prepared by coimpregnation technique and microwave-drying method showed enhanced catalytic activities for selective dehydrogenation of *n*-dodecane, who has no double bonds to its corresponding mono-olefins and preferential oxidation of CO in the presence of excess H_2_ [56,57]. Binary Cr–Mo oxide catalysts supported on MgO-coated polyhedral three-dimensional mesoporous SBA-16 have been prepared for the oxidative dehydrogenation of iso-butane [58].

Yang *et al*. [59] prepared an efficient and highly recyclable heterogeneous catalyst for the Suzuki and Heck reactions by grafting an *N*-heterocyclic carbene palladium complex and ionic liquid on the mesoporous cage-like material SBA-16.

On the other hand, upgrading olive oil production by-products via hydrotreating was performed on sulfided CoMo catalysts supported on SBA-16 [60]. Dong *et al*. [61] prepared CuO/SBA-16 catalysts by a modified impregnation method. This kind of catalyst has a dispersed CuO which is considered as highly efficient species for hydroxylation of phenol with H_2_O_2_. Zhu *et al*. [62] reported that SBA-16 supported vanadium oxide catalyst showed good catalytic performance for the direct hydroxylation of benzene to phenol by hydrogen peroxide under moderate condition.

Recently, Elangovan *et al*. [63], carried out the catalytic transformation of 1-adamantanol over sulfonic-acid functionalized SBA-16 and Tsoncheva *et al*. [64] reported the incorporation of nanosized iron oxide particles within SBA-16 material and their use as catalyst for the methanol decomposition to H_2_, CO and methane.

### 4.2. Functionalization

Shortly after the discovery of ordered SBA-16 mesoporous silica, scientists started to explore the possibility of modifying the surface properties of such material by modifying it with organic groups. Wang, *et al*. [65] reported that a vinyl-functionalized analogous to SBA-16 can be synthesized by co-condensation of tetraethoxysilane (TEOS) and triethoxyvinylsilane (TEVS) in the presence of the triblock copolymer Pluronic P123 and inorganic salts such as NaCl. Leisant *et al*. [66] reported the synthesis of SBA-16 with mercaptopropyl groups by two routes: post synthesis grafting of pure mesoporous silica and direct functionalization by a co-condensation procedure.

Grudzien *et al*. [67–69] and Wei *et al*. [70] demonstrated a successful incorporation of bulky isocyanurate bridging groups into the cage-like silica framework of *Im*3*m* symmetry and amino-functionalized SBA-16 prepared by silylating various *N*-(2-aminoethyl)-3-aminopropyltrimethoxysilane (AEAPS) contents. In this respect, Knöfel *et al*. [71] and Han *et al*. [72] studied the functionalization of SBA-16 support with the diamine (CH_3_O)_3_Si-(CH_2_)_3_-NH-(CH_2_)_2_-NH_2_ by post-synthesis grafting and by using a cobalt diaminosarcophagine cage complex covalently grafted onto the silica surface through the silication with sylanol group, respectively.

On the other hand, Ueno *et al*. [73] incorporated aromatic carboxylic acid molecules in SBA-16 and Sujandi *et al*. [74] studied chloropropyl-functionalized mesoporous silica with cage-type cubic Im3m phases.

### 4.3. Metals Incorporation

The synthesis of metal-substituted and metal-containing SBA-16 mesoporous materials has attracted considerable interest for potential applications mainly in the catalysis field. Al-SBA-16, Ti-SBA-16, V-SBA-16, Fe-SBA-16 and Nb-SBA-16 mesoporous silicas with a cubic Im3m structure have been successfully synthesized using silica precursors and a triblock copolymer under acidic condition [44,75–80].

In the case of Ti, the synthesis through prehydrolysis of a silica precursor in the presence of a triblock copolymer F127 under acidic conditions [76] and by an evaporation-induced self-assembly method using F127 copolymer as template have been reported [77]. In both cases the Ti loading has been increased up to high values (~15 wt%) and titanium species were highly dispersed in the silica framework with tetrahedral and octahedral coordination. These materials possesses high thermal stability, thick pore walls, and high surface area with a mesoporous worm-like structure, showing high activity in the oxidative desulfurization of DBT that was not reduced even after recycling several times.

Fe-SBA-16 with isolated framework Fe species was synthesized by simple adjustment of the molar ratio of co-surfactant *n*-butanol, TEOS, *n*_H2O_/*n*_HCl_ ratio, Si/Fe ratio, and aging conditions, respectively [79]. It was reported that the presence of both framework and extra-framework species in the catalysts is related to the cyclohexene conversion and 2-cyclohexene-1-one activity and selectivity.

Nb-SBA-16 was obtained under acidic conditions and the pore diameters were tuned by varying the hydrothermal treatment temperature and time. It was found that pore volume, pore diameter, and micro-/mesopores ratio can be controlled very efficiently by changing the synthesis parameters [80].

Loading gold on mesoporous materials via different methods has been actively attempted in the literature, but the knowledge about the influences of synthesis details and different mesoporous structures on the size and thermal stability of gold nanoparticles supported on mesoporous hosts is still limited. Glomm *et al*. [81] and Lee *et al*. [82] reported the incorporation of gold on SBA-16 material using different strategies and Shi *et al*. [83] studied the addition of Fe_2_O_3_ nanowires. In addition, NiO, CoO, CoMoO_4_, MoO_3_, Cr-Mo, WC_x_, WO_3_ and Pt-Sn [45–47,57,58] species have been incorporated to SBA-16 material.

### 4.4. Environmental

Due to the control both the size and the shape of the pores in the structure of SBA-16, this material has excellent potential for application in the environmental area, although it has been little exploited. Polyethyleneimine (PEI)-loaded mesoporous silica materials using SBA-16 have been developed in order to evaluate their performances in terms of CO_2_ adsorption, showing reversible CO_2_ adsorption–desorption behavior with >99% recovery [84]. Related to this, the CO_2_ adsorption capacity of SBA-16 functionalized with *N*-(2-aminoethyl)-3-aminopropyltrimethoxysilane (AEAPS) was evaluated and the effects of hydrolysis and particle size on functionalisation of the SBA-16 support were investigated [85].

In another vein, functionalized SBA-16 mesoporous silica with –SH groups was employed as a Cu(II) ion adsorbent from aqueous solutions at room temperature and it has been reported that by controlling an optimum molar ratio between tetraethyl orthosilicate (TEOS) and 3-mercaptopropyltriethoxysilane (TMMPS), used as organic functionality agent, the obtained material possessed high order and adsorption capacity for Cu(II) ions, probably due to the adsorption through ligand exchange with the –SH group [86]. Another reported application corresponds to the catalytic potential of chloroperoxidase (CPO) immobilized on mesoporous materials for the oxidation of 4,6-dimethyldibenzothiophene in water/acetonitrile mixtures [87]. It has been found that the nature and the pore size of the material does affect the catalytic activity of the enzyme and its stability.

### 4.5. Template

The use of the structure of SBA-16 as a template has gained considerable interest in recent years, this due to their characteristics, such as control of the morphology, pore size, ordered pore structure, high surface area, large pore volume, etc. For example, continuous thin films of SBA-16, prepared on indium-tin oxide glass (ITO) via dip-coating technique, has been used as a template to produce three-dimensional porous crystals of iron oxide by electrochemical deposition of iron metal followed by in-situ oxidation [88].

Mesoporous carbon materials as well as mesoporous carbon-nitride-based hybrid material (MCN-2) with very high surface area, pore volume, and a possible cage type porous structure has been prepared by using mesoporous SBA-16 silica as a hard template [89–91]. In the first two cases, it has been found that surfactant removal method plays an important role in the formation of carbon mesostructures; in the last one, authors report that because of the excellent textural characteristic and three-dimensional porous structure, MCN-2 material could offer great potential for the applications in catalysis and adsorption.

In other hand, several mesoporous metal oxides have been prepared by using the SBA-16 structure as template. Yue *et al*. [92] reported the obtention of mesoporous single-crystal Co_3_O_4_ by using cagecontaining mesoporous silicas, such as SBA-16, as templates.

Solvent-free infiltration method was used as a simple and facile way for the preparation of mesoporous metal oxide materials via nano-replication from mesoporous SBA-16 templates, and mesoporous tin oxide (SnO_2_) materials was reported by korean researchers [93].

Following this line of research, the synthesis of large specific surface area LaFeO_3_ nanoparticles by SBA-16 template method has been recently reported [94]. The material was obtained by impregnation process with corresponding metal nitrates as La and Fe resources and it was found that this material has better structural properties compared with those made by conventional citrate method and superior photocatalytic activity in the degradation of Rhodamine B solution under visible irradiation than P25 TiO_2_. This excellent photocatalytic performance is mainly attributed to the large surface area and high photogenerated charge separation rate. It should be noted that mesoporous single-crystal oxides, prepared by these methods, may be developed into new forms of catalysts with high activity and selectivity, and new materials with some interesting physical properties.

Another solvent-free nanocasting route reported, by using using periodic mesoporous SBA-16 silicas, corresponds to the synthesis of periodic mesoporous phosphorus–nitrogen (P-N) frameworks with cubic symmetry [95]. The precursor (PNCl_2_)_3_ was molten at 120 °C in nitrogen atmosphere and infiltrated into the pores of mesoporous silica, followed by nitridization in an ammonia atmosphere and finally by the SiO_2_ template removal. The results support the idea that this nanocasting procedure is a promising pathway to the obtention of these compounds even if the chemistries of the hard template and the target material are very similar and can react easily with each other.

### 4.6. Electronics

In the field of electronics, SBA-16-based materials have been used in different ways, such as surface photo voltage (SPV) sensors for NO and NO_2_ gases [96,97]. SBA-16-type mesoporous silica films were fabricated in a metal–insulator–semiconductor (MIS) device and this mesoporous SPV system exhibits a recoverable response much larger than that of a simple SPV sensor without mesoporous silica film; *i.e.* the cubic-like mesoporous silica film improves the sensing characteristics of the SPV gas sensor due to the physical adsorption of the target gas into the mesoporous layer, which is regarded as the insulator layer in metal-insulatorsemiconductor (MIS) devices. Authors attribute this result to the large surface area and a bi-continuous mesopore structure of the cubic-like structure of SBA-16. Another application in this direction corresponds to the use of these highly ordered mesoporous silicates in the fabrication of sensing microfluidic devices of environmental pollutant gases, achieving high selectivity with respect to benzene, toluene and xylene (BTX), even in a pseudo-atmospheric environment [98].

In other hand, mesostructured SBA-16 silica materials served as templates used to obtain mesoporous carbons for use as electrodes for electrochemical capacitors. These templated mesoporous carbons with tailored pore size distribution are very promising materials to be used as electrodes in supercapacitors [99].

Low-dielectric constant (low-κ) polyimide (PI) composite films containing the SBA-16-type mesoporous silica were prepared via in situ polymerization and a reduction in the dielectric constant is reported besides an improvement of the thermal stability and dynamic mechanical properties of the PI film due to the incorporation of the mesoporous silica materials [100]. Brominated epoxy resin (BER) composites containing various amounts of SBA-16 types mesoporous silicas, prepared through the thermal curing with 3-methyltetrahydrophthalic anhydride, as well as *o*-cresol novolac epoxy (*o*-CNER)-based composites, have also been evaluated as low-κ materials [101,102].

SBA-16, among other ordered mesoporous silica host materials, functionalised with sulfonic acid groups were obtained by anchoring and oxidation of thiol groups. The proton conductivity was evaluated and concluded that mesoporous material based highly acidic composites are good candidates for an application in fuel cell membranes being the proton conductivity depends on the texture of the host system [103].

Manning *et al*. [104] mention that the use of periodic nanostructures, such as SBA-16, has opened up the possibility of generating periodic magnetic nanostructures of types not accessible by self-assembly of nano-particles and that such materials can be fabricated on a wide range of metal substrates.

Recently, Tu *et al*. [105] has reported the use of mesoporous silica SBA-16 doped with Li as humidity sensor material, showing significant change in impedance of more than three orders of magnitude over almost the whole humidity range (11–95% RH), with fast response time (about 25 s), and relatively low hysteresis (about 4%). In summary, this kind of mesoporous material has a great potential for application to highly sensitive and responsive sensors.

## 5. Conclusions and Future Prospects

**Figure 3:**
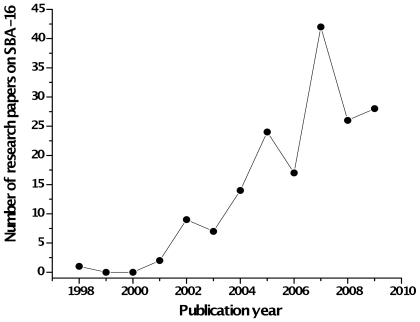
Evolution of the number of research papers on SBA-16 published by year.

Both the synthesis, as well the use and applications of materials based on the SBA-16 have resulted in an increasing number of international publications. Figure 3 shows the number of research articles based on SBA-16 published each year and cited in this work.

As shown, there is a very clear upward trend and is expected to increase in coming years when more applications are developed for this material. This is due to the ease of the method of preparation, to the orderly structure, size and shape of their pores and control all these through simple changes in the method of synthesis, so that the SBA-16 has become a very versatile material, potentially applicable in many areas of science and molecular engineering of materials. Compared with other silica mesoporous materials as such as MCM-41 and MCM-48, SBA-16 materials show important advantages like thicker framework walls, small crystallite size of primary particles, super-large cage, complementary textural porosity, higher thermal stability and more favorable mass transfer than the unidirectional pore system of hexagonal mesoporous materials due to three-dimensional channel connectivity. The later property is very important since SBA-16 minimize the mass transfer problems of bulky reactants and products, which may be encountered in other mesoporous materials. The importance that has taken this material can be illustrated taking into account the number of international publications per year by taken as the reference articles that only appear in the indexed database SCOPUS ™ associated with SBA-16.

This underlines the importance of a revision of the state of the art in this issue, to take stock of knowledge about the methods of synthesis, characterization techniques and applications of this material to develop new materials and novel applications with interesting physical and chemical properties.
